# Clinical efficacy of different maintenance doses of caffeine citrate in the treatment of apnea of prematurity

**DOI:** 10.12669/pjms.41.2.9692

**Published:** 2025-02

**Authors:** Ping Ji, Yu Wan, Jiansong Yin, Mei Xue, Jing Wang, Liwen Zhang

**Affiliations:** 1 Ping Ji Neonatal Intensive Care Unit, Second People’s Hospital of Changzhou Affiliated to Nanjing Medical University, Changzhou 213164, Jiangsu, China; 2 Yu Wan Neonatal Intensive Care Unit, Second People’s Hospital of Changzhou Affiliated to Nanjing Medical University, Changzhou 213164, Jiangsu, China; 3 Jiansong Yin Neonatal Intensive Care Unit, Second People’s Hospital of Changzhou Affiliated to Nanjing Medical University, Changzhou 213164, Jiangsu, China; 4 Mei Xue Neonatal Intensive Care Unit, Second People’s Hospital of Changzhou Affiliated to Nanjing Medical University, Changzhou 213164, Jiangsu, China; 5 Jing Wang Child Healthcare Department, Second People’s Hospital of Changzhou Affiliated to Nanjing Medical University, Changzhou 213164, Jiangsu, China; 6 Liwen Zhang Pediatric Department, Second People’s Hospital of Changzhou Affiliated to Nanjing Medical University, Changzhou 213164, Jiangsu, China

**Keywords:** Citrate Caffeine, Preterm Infant, Apnea, Clinical Efficacy, Cost Effect

## Abstract

**Objective:**

To investigate the efficacy and adverse reactions of caffeine citrate in the treatment of primary apnea of prematurity(AOP) and its effect on the long-term development of preterm infants.

**Methods:**

This was a retrospective study. One hundred and forty six infants with AOP treated in the Neonatal Department of Second People’s Hospital of Changzhou Affiliated to Nanjing Medical University between December 2019 and December 2023 were divided into the low-dose group (5mg/kg) and the high-dose group (10 mg/kg) according to the maintenance doses of caffeine, with 73 patients each group. Efficacy, complications, adverse reactions, total hospitalization cost and long-term development of the two groups of patients were retrospectively compared.

**Results:**

Short-term efficacy: No statistically significant differences in the number of apnea events, assisted ventilation duration, oxygen inhalation duration, and weaning success rate were observed between the two groups (*P*<0.05). The incidences of bronchopulmonary dysplasia (BPD) (*P*=0.012) and periventricular leukomalacia (PVL) (*P*=0.005) in the high-dose group were decreased compared with those in the low-dose group, with statistically significant differences. Long-term efficacy: the motor and mental development scores at 12 months of age in the high-dose group were increased compared with those in the low-dose group (*P*<0.05); and cost-effect analysis: there was no significant difference in the total hospitalization cost between the two group (*P*>0.05).

**Conclusion:**

High maintenance dose of citrate caffeine in the treatment of AOP significantly reduces the number of apnea events, oxygen inhalation duration, the incidence of BPD and PVL, and positively affects the long-term neurological and motor development, without a significant increase in the hospitalization cost.

## INTRODUCTION

Apnea of prematurity (AOP) is common in infants with a gestational age of less than 34 weeks. It is defined as a pause of breathing for more than 20 seconds or a pause of less than 20 seconds accompanied by a slow heart rate or cyanosis. Recurrent episodes of apnea can lead to hypoxic damage and even preterm infant death.^1^ The main cause of AOP is the immature respiratory center of infants. Caffeine is a non-specific adenosine receptor antagonist that can be used as a central nervous system stimulant^2^, and has been clinically used to treat AOP since introduced in China in 2013. Currently, this drug can be administered intravenously or orally, with a longer half-life, and can be administered once a day.^3^ Caffeine citrate was first used in the neonatal intensive care unit in Second People’s Hospital of Changzhou Affiliated to Nanjing Medical University in 2016 to treat AOP in preterm infants, this study was to investigate the efficacy and adverse reactions of caffeine citrate in the treatment of primary apnea of prematurity(AOP) and its effect on the long-term development of preterm infants.

## METHODS

This was a retrospective study. December 2019 and December 2023 preterm infants were diagnosed with primary apnea in the Neonatal Department of Second People’s Hospital of Changzhou Affiliated to Nanjing Medical University between December 2019 and December 2023 were divided into the low-dose group (n=73) and the high-dose group(n=73) according to the maintenance doses of caffeine. There were 77 males and 69 females, with a gestational age of 26+4 weeks to 33+5 weeks, and a birth weight of 1000-1800g. These infants were transferred from the obstetric department to the neonatal department immediately after birth.

### Ethical Approval:

The study was approved by the Institutional Ethics Committee of Second People’s Hospital of Changzhou Affiliated to Nanjing Medical University(No.:[2023]KY114-01; date: July 20, 2023), and written informed consent was obtained from all participants’ guardians.

### Inclusion criteria:

- Preterm infants diagnosed with primary apnea.
- With a gestational age of 26+4 weeks to 33+5 weeks.
- A birth weight of 1000-1800 g.

### Exclusion criteria:

- Infants with congenital malformation (such as complex congenital heart disease, tracheopulmonary malformation, and digestive tract malformation).
- Severe infections (such as septicemia and suppurative meningitis).
- Genetic metabolic diseases.
- Obstructive apnea.
- Admitted to the hospital six hours after birth and discharged voluntarily.

All children received assisted ventilation (including ventilatory assist ventilation, continuous positive airway pressure[CPAP] ventilation or hood, and nasal cannula oxygen therapy) after the defined diagnosis of AOP, and other treatments were given to maintain homeostasis. Nutritional support was provided, and infections were prevented and treated.

### High-dose caffeine maintenance treatment:

Caffeine citrate was given in addition to the basic treatment for patients in the high-dose group. Caffeine citrate was administered according to the European Guidelines for the Prevention and Treatment of Neonatal Respiratory Distress Syndrome, 2016 edition. A loading dose of 20 mg/kg/d was given intravenously over 30 minutes, and 24 hours later, a maintenance dose of 10 mg/kg/d was given. When the children tolerated full gastrointestinal feeding, intravenous administration of the caffeine citrate was changed to oral administration, with the same dosage and time as intravenous administration. The drug was discontinued till the corrected gestational age of 34 weeks with no obvious apnea.^4^ If patients experienced apnea during this period, plantar stimulation, back support, and facemask pressure ventilation with a gasbag were provided, and mechanical ventilation should be provided if necessary.

### Low-dose caffeine maintenance treatment:

In addition to basic treatment, caffeine citrate was intravenously given at a loading dose of 20 mg/kg/d over 30 minutes, followed by a maintenance dose of 5 mg/kg/d 24 hours later. *Drugs* Caffeine citrate injection (Peyona^®^), specification: 20 mg/1 ml per vial, batch number 20130109, produced by Chiesi Farmaceutici SpA, Italy.

### Outcome measures of short-term efficacy:

The number of apnea events: the number of apnea events in three days of treatment; assisted ventilation duration: duration of invasive and non-invasive ventilation; oxygen inhalation duration: the time of oxygen inhalation after ventilators were removed; and weaning failure: unable to remove the ventilator or reuse it within 24 hours after removal.

### Evaluation of complications and adverse reactions:

BPD, severe retinopathy of prematurity(ROP), PVL, PDA requiring surgical treatment, and liver and renal dysfunctions. Related diagnostic criteria referred to Practical Neonatology, 5th edition.^5^ Poor weight gain: Weight gain was less than 20 g/kg/d; feeding intolerance^6^:

- the number of vomiting was ≥ 3 times/day;
- feeding volume did not increase or decrease for more than three consecutive days,
- gastric retention was > 1/3 of the previous feeding volume; and tachycardia in preterm infants: The heart rate was greater than 180 times/minutes.^7^

### Outcome measures of long-term efficacy:

The motor and mental development scores were assessed at 12 months of age using the Bayley scales of Infant and Toddler Development. The total hospitalization cost(in RMB yuan).

### Statistical analysis:

Data were analyzed using SPSS 26.0 software. Measurement data with normal distribution were presented as , and an independent sample t test was used for comparison between groups; those with non-normal distribution were presented as M (P 25, P 75), and the rank sum test was used for comparison between groups. Numeration data were presented as n(%), and the chi-square test was used for comparison between groups. Fisher exact probability test was used if the applicable conditions were not met. Differences with a p-value of <0.05 were considered statistically significant.

## RESULTS

There were no statistically significant differences between the two groups of children in terms of sex, gestational age, birth weight, five minutes Apgar score, delivery mode, assisted ventilation, and use of pulmonary surfactant (P>0.05) (Table-I).

**Table-I:** Comparison of general data between two groups of infants[*χ̅*±*S* or n(%)].

Groups	n	Sex		Gestational age (weeks)	Birth weight (g)	5-minute Apgar score (points)	Delivery mode[n(%)] Normal labor	Mechanical ventilation or CPAP ventilation [n(%)]	Use of PS [n(%)]

		M	F						
The high-dose group	73	38 (52.1)	35 (46.0)	30.58±1.79	1269.59±190.56	7.97±1.09	44 (60.3)	67 (91.8)	52 (71.2)
The low-dose group	73	39 (53.4)	34 (49.2)	30.74±1.82	1251.78±149.74	7.89±0.98	46 (63.0)	65 (89)	54 (74.0)
*χ*^2^/t		0.027		-0.542	0.628	0.479	0.116	0.316	0.138
*p*		0.868		0.589	0.531	0.633	0.734	0.574	0.711

***Note:*** CPAP, continuous positive airway pressure; PS, pulmonary surfactant.

The number of apnea events assisted ventilation duration, and oxygen inhalation duration were decreased, and the weaning success rate was increased in the high-dose group compared with those of the low-dose group, respectively, and the differences were statistically significant(P<0.05) (Table-II).

**Table-II:** Comparison of short-term efficacy between the two groups [*χ̅*±*S* or n(%)].

Groups	n	Number of apnea events in 3 days of treatment(times)	assisted ventilation duration(days)	oxygen inhalation duration(days)	Weaning failure[n(%)]	Hospital stay(days)
The high-dose group	73	12.23±4.16	10.34±8.97	5.23±3.95	4(5.5)	40.30±13.51
The low-dose group	73	16.79±4.76	14.38±5.87	8.41±7.53	13(17.8)	44.70±14.52
*χ*^2^/t		-6.164	-2.286	-3.194	5.393	-1.894
*p*		<0.001	0.024	0.002	0.020	0.060

The incidences of BPD and PVL in the high-dose group decreased compared with that in the low-dose group, with statistically significant differences(P<0.05), and no statistically significant differences in the incidences of ROP, NEC, and PDA were observed between the two groups(P>0.05) (Table-III). There were no statistically significant differences in the incidences of tachycardia, feeding intolerance, poor weight gain, liver dysfunction, and renal dysfunction between the two groups of preterm infants(P>0.05) (Table-IV). There was no statistically significant difference in hospitalization cost between the two groups(P>0.05) (Table-V).

**Table-III:** Comparison of complications between the two groups[n(%)].

Groups	n	BPD[n(%)]	ROP[n(%)]	PVL[n(%)]	NEC[n(%)]	PDA[n(%)]
The high-dose group	73	4(5.5)	4(5.5)	3(4.1)	3(4.1)	8(11)
The low-dose group	73	14(19.2)	6(8.2)	14(19.2)	3(4.1)	10(13.7)
*χ* ^2^		6.337	0.429	8.056	—	0.253
*p*		0.012	0.512	0.005	1.000*	0.615

***Note:*** BPD, bronchopulmonary dysplasia; ROP, retinopathy of prematurity; PVL, periventricular leukomalacia; NEC, necrotizing enterocolitis; PDA, patent ductus arteriosus; —, Fisher’s exact probability method was used.

**Table-IV:** Comparison of adverse reactions between the two groups.

Groups	n	Tachycardia [n(%)]	Feeding intolerance [n(%)]	Poor weight gain [n(%)]	Liver dysfunction [n(%)]	Renal dysfunction [n(%)]
The high-dose group	73	13(17.8)	17(23.3)	3(4.1)	5(6.8)	3(4.1)
The low-dose group	73	8(11)	15(20.5)	5(6.8)	6(8.2)	4(5.5)
*χ* ^2^		1.390	0.160	0.529	0.098	—
*p*		0.238	0.689	0.467	0.754	1.000*

***Note:*** —, Fisher’s exact probability method was used.

**Table-V:** Comparison of hospitalization cost between the two groups.

Groups	Total hospitalization cost	t	P
The high-dose group (n=73)	57779.38(40479.72, 78737.91)	-1.500	0.133
The low-dose group (n=73)	51456.64(33657.21, 68415.58)		

The scores of motor and mental development at 12 months of age were improved in the high-dose group compared with those in the low-dose group, and the differences were statistically significant(P<0.05) (Table-VI).

**Table-VI:** Comparison of the long-term motor and mental developments between the two groups.

Items	n	At 12 months of age	

		MDI score	PDI score
The high-dose group	70	99(92.00, 110.00)	97(85.00, 109.00)
The low-dose group	69	89.5(82.00, 101.75)	91(81.00, 102.15)
*t*/*z*		-4.321	-2.945
*P*		<0.001	0.004

***Note:*** MDI, mental development index; PDI, psychomotor development index.

## DISCUSSION

The present study found that when caffeine was used for long-term maintenance therapy in preterm infants in their early postnatal period, durations of ventilation and oxygen inhalation were significantly shortened, and the incidence of BPD was significantly reduced in the high-dose group compared with those in the low-dose group, respectively, with no significant adverse reactions found. Caffeine citrate can protect the nervous system. It has been found that caffeine can improve the hypoxia and ischemia of brain cells, participate in the anti-inflammatory effect, reduce brain damage caused by oxidative stress, promote the myelination development of white matter, and enhance brain electrical activity.^8^ Studies have shown that the incidence of adverse neurological prognosis and cerebral palsy in the caffeine group was decreased compared with that in the placebo group at 18 months after the corrected gestational age in preterm infants with a birth weight of less than 1250g who were treated with caffeine from three days after birth to 34 weeks of corrected gestational age.^9^ DeMauro SB showed that caffeine treatment significantly reduced the incidence of cerebral palsy and delayed cognitive development.^10^ Yang L et al.^11^ found that the neurological development of preterm infants with AOP was significantly improved at six months in the caffeine treatment group compared with that in the aminophylline treatment group, with a significant repair effect on white matter damage(WMD). WMD was significantly improved in the caffeine group at 40 weeks of corrected gestational age, and 40 weeks of corrected gestational age was significantly correlated with six months neurodevelopment.

Studies have also shown that the maturity of white matter microstructure and the volume of white matter in the treatment group were increased compared with those in the control group in preterm infants treated with caffeine citrate for a long period of time in the early stage as evidenced by head MRI diffusion tensor imaging.^12^ It was found in the present study that the incidence of PVL in the high-dose group was decreased compared with that in the low-dose group, indicating a stronger brain protective effect of the high-dose caffeine in the maintenance treatment. The motor and mental development scores of the high-dose group were increased compared with those of the low-dose group at 12 months of age, and it was considered that maintenance treatment with a high dose of caffeine can improve the long-term prognosis of preterm infants.

The mechanism of primary apnea in preterm infants is currently unclear, which possibly involves the low degree of neuronal myelination in the respiratory center, limited number of synaptic and dendritic connections, and incomplete and untimely signal transmission in these infants. Therefore, the transmission of neural impulses in tissues and the nervous system is weak.^13^ Repeated, persistent, and frequent episodes of apnea easily lead to hypoxic brain injury and even death if not treated actively and effectively.^14^ The main medical treatments currently available include methylxanthines, with theophylline, aminophylline and caffeine as representatives, and the underlying mechanism of action of these drugs is to stimulate the respiratory system by antagonizing peripheral and central adenosine A1 and A2 receptors.^15^

Blocking of these receptors can eliminate the inhibition of respiratory adenosinergic effects by the medullary respiratory control area in the central nervous system(CNS), prevent the release of γ-aminobutyric acid, and increase nerve excitability and respiratory drive.^16^,^17^ Kou et al.^18^ conducted a study in 56 premature infants with a body weight of ≤ 1500 g and a gestational age of <32 weeks, and found that prophylactic treatment with caffeine reduced the levels of inflammatory factors and the incidence of BPD in these infants. Caffeine citrate can promote successful ventilation weaning, shorten the oxygen inhalation duration, reduce lung inflammation, improve airway remodeling, and consequently prevent the development of BPD via improving lung compliance, reducing airway resistance, enhancing diaphragm contraction, and increasing minute ventilation.^8^ Wan L et al.^19^ showed that high-maintenance doses of caffeine (10 mg/kg) can shorten hospital stay and oxygen inhalation duration without increasing the incidence of adverse reactions.

Studies have shown that caffeine can block early apoptosis and promote the normal differentiation of oligodendrocyte precursor cells by antagonizing adenosine A1 receptors to alleviate PVL injury and improve the structural development of white matter in the brain of preterm infants, suggesting the presence of other unknown mechanisms involved in the cerebral protective effect of caffeine citrate.^20^,^21^ No difference in liver and renal dysfunctions was noted between the two groups in the present study, indicating that the incidence of adverse drug reactions did not increase when a high-maintenance dose of caffeine was used.

Caffeine citrate commonly used in most hospitals in China is produced by Chiesi Farmaceutici SpA, Italy. It is an imported drug with a relatively high price. No statistically significant difference in the total hospitalization cost was observed between the high-dose group and the low-dose group, indicating that a high-maintenance dose of caffeine is not associated with a significant increase in hospitalization costs. This may be explained by the longer ventilation and oxygen inhalation duration in the low-dose group, which increases the treatment cost. From this point of view, maintenance therapy with a high dose of caffeine should be recommended.

### Limitations:

However, this study also has some shortcomings, the small sample size, which may lead to result bias. Future studies with a larger sample size are needed to confirm the long-term development findings.

## CONCLUSIONS

The present study suggested that a high-maintenance dose of caffeine is more effective in the treatment of primary AOP in preterm infants, and positively affects the long-term development of these infants, with no significantly increased side effects and hospitalization costs.

### Authors’ Contributions:

**PJ** and **YW:** Carried out the studies, participated in collecting data, and drafted the manuscript, and are responsible and accountable for the accuracy or integrity of the work.

**JY, JW, LZ** and **MX:** Performed the statistical analysis and participated in its design.

All authors have read and approved the final manuscript.
